# The Global Burden of Maxillofacial Trauma in Critical Care: A Narrative Review of Epidemiology, Prevention, Economics, and Outcomes

**DOI:** 10.3390/medicina61050915

**Published:** 2025-05-18

**Authors:** Antonino Maniaci, Mario Lentini, Luigi Vaira, Salvatore Lavalle, Salvatore Ronsivalle, Francesca Maria Rubulotta, Lepanto Lentini, Daniele Salvatore Paternò, Cosimo Galletti, Massimiliano Sorbello, Jerome R Lechien, Luigi La Via

**Affiliations:** 1 Department of Medicine and Surgery, University of Enna “Kore”, 94100 Enna, Italy; antonino.maniaci@unikore.it (A.M.); mario.lentini@unikore.it (M.L.); salvatore.lavalle@unikore.it (S.L.); salvatore.ronsivalle@unikore.it (S.R.); lepanto.lentini@unikore.it (L.L.); cosimo.galletti01@unikore.it (C.G.); massimiliano.sorbello@unikore.it (M.S.); 2 ASP Ragusa, 97100 Ragusa, Italy; paternomd@icloud.com; 3 Study Group of Young-Otolaryngologists of the International Federations of Oto-Rhino-Laryngological Societies (YO-IFOS), 75001 Paris, France; luigi.vaira@gmail.com (L.V.); jerome.lechien@umons.ac.be (J.R.L.); 4 Maxillofacial Surgery Unit, Department of Medicine, Surgery and Pharmacy, University of Sassari, 07100 Sassari, Italy; 5 Department of General Surgery and Medical-Surgical Specialties, CHIRMED University of Catania, 95123 Catania, Italy; francesca.rubulotta@unict.it; 6 Department of Anesthesia and Intensive Care 1, University Hospital Policlinico “G. Rodolico-San Marco”, 95123 Catania, Italy; 7 IWIN Foundation, 95100 Catania, Italy; 8 Department of Anatomy and Experimental Oncology, Mons School of Medicine, UMONS Research Institute for Health Sciences and Technology, University of Mons (UMons), 75001 Mons, Belgium

**Keywords:** maxillofacial trauma, facial injuries, prevention strategies, road safety measures, economic burden, surgical management, public health impact

## Abstract

*Background and Objectives*: Maxillofacial trauma represents a significant global health challenge with substantial physical, psychological, and socioeconomic consequences. *Materials and Methods:* This narrative review analyzed 112 articles published between 2000 and 2024 examining epidemiology, prevention, economics, and outcomes of maxillofacial trauma in critical care settings. *Results*: Road traffic accidents remain the primary cause globally, followed by interpersonal violence and occupational injuries. Effective prevention strategies include seat belt laws, helmet legislation, and violence prevention programs. Economic burden encompasses direct healthcare costs (averaging USD 55,385 per hospitalization), productivity losses (11.8 workdays lost per incident), and rehabilitation expenses (USD 3800–18,000 per patient). Surgical management has evolved toward early intervention, minimally invasive approaches, and advanced techniques using computer-aided design and 3D printing. Complications affect 3–33% of patients, with significant functional disabilities and psychological sequelae (post-traumatic stress disorder in 27%, depression/anxiety in 20–40%). *Conclusion*: Maxillofacial trauma management requires multidisciplinary approaches addressing both immediate treatment and long-term rehabilitation. Despite technological advances, disparities in specialized care access persist globally. Future efforts should implement evidence-based prevention strategies, reduce care disparities, and develop comprehensive approaches addressing physical, psychological, and socioeconomic dimensions through collaboration among healthcare professionals, policymakers, and community stakeholders.

## 1. Introduction

Maxillofacial trauma represents a significant global health challenge, encompassing injuries to facial soft and hard tissues that can profoundly impact critical functions like breathing, eating, and speaking [1,2]. The worldwide incidence of these injuries places substantial strain on healthcare systems across different socioeconomic settings [3]. Despite its significance, there exists a notable gap in the comprehensive understanding of maxillofacial trauma’s global burden, particularly within critical care contexts.

Road traffic accidents (RTA) remain the primary cause of maxillofacial fractures, particularly in low- and middle-income countries [4], though other significant causes include interpersonal violence, falls, sports injuries, and industrial accidents [5]. The distribution and patterns of these injuries exhibit marked geographical variation, reflecting differences in socioeconomic conditions, cultural factors, and legislative environments [6]. This variability presents challenges for developing universally applicable management protocols and prevention strategies.

The management of maxillofacial trauma demands a multidisciplinary approach [7], with immediate priorities including airway maintenance, hemodynamic stabilization, and hemorrhage control [8,9]. Beyond the acute phase, these injuries often result in significant functional, aesthetic, and psychological consequences [10,11,12], while also imposing substantial economic burdens through direct healthcare costs and lost productivity [13,14]. The economic dimensions of maxillofacial trauma remain insufficiently characterized in the literature, presenting an important area for further investigation.

Prevention strategies are crucial in reducing the global burden of maxillofacial trauma. Traditional preventive measures include road safety regulations [15] and public awareness campaigns [16]. However, as highlighted by Siegler and Rogers in their comprehensive analysis of trauma systems, environmental and social factors play a crucial role in trauma prevention. Their work demonstrates how socioeconomic disparities significantly influence both the incidence and outcomes of trauma, particularly in underprivileged areas. This suggests that addressing underlying social determinants is equally important for effective prevention.

Recent advances in imaging, surgical techniques, and biomaterials have transformed treatment approaches for maxillofacial trauma [17,18,19], though significant disparities in access to specialized care persist, particularly in developing regions [20,21]. The impact of these technological advances on outcomes in critical care settings has not been systematically evaluated.

This review aims to address several key research questions: What is the current global epidemiological landscape of maxillofacial trauma requiring critical care? What are the most effective prevention strategies across different socioeconomic settings? What is the economic impact of maxillofacial trauma on healthcare systems and society? How do management approaches and outcomes vary across healthcare settings? By addressing these questions, we seek to bridge important knowledge gaps in the understanding of maxillofacial trauma in critical care settings and inform future research directions for developing novel treatment strategies, refining outcome assessments, and implementing evidence-based prevention programs [22,23].

## 2. Methods

### 2.1. Search Strategy

We conducted a comprehensive literature search using multiple electronic databases including PubMed/MEDLINE, Embase, Scopus, and Google Scholar for articles published between January 2000 and September 2024. The following specific keyword combinations were used:

“maxillofacial trauma” OR “facial trauma” OR “facial injuries” OR “facial fractures”

AND “critical care” OR “intensive care” OR “ICU” OR “trauma center”

AND “epidemiology” OR “incidence” OR “prevalence” OR “demographics”

AND “economics” OR “cost” OR “financial burden” OR “resource utilization”

AND “prevention” OR “intervention” OR “safety measures”

AND “outcomes” OR “complications” OR “mortality” OR “morbidity”

### 2.2. Selection Criteria

Articles were selected based on their relevance to maxillofacial trauma management in critical care settings. We included original research articles, review articles, and clinical guidelines published in English. Studies addressing pediatric populations exclusively were excluded to maintain focus on adult critical care. Case reports were included only if they described novel management approaches or rare complications relevant to critical care.

### 2.3. Data Extraction and Analysis

We initially identified 427 potentially relevant articles. After removing duplicates and screening titles and abstracts, 183 articles were selected for full-text review. Finally, 112 articles met our inclusion criteria and were included in this review. From these articles, we extracted data on the following:

- Epidemiological patterns (incidence, prevalence, demographic characteristics);
- Etiology and mechanisms of injury;
- Prevention strategies and their effectiveness;
- Economic impact (direct healthcare costs, productivity losses, and rehabilitation expenses);
- Management approaches in critical care settings;
- Short-term and long-term outcomes;
- Complications specific to maxillofacial trauma in critical care.

The extracted information was organized thematically to identify patterns, trends, and gaps in current knowledge. Particular attention was given to geographical variations and differences between high-income versus low- and middle-income countries.

## 3. Epidemiology

**Figure 1:**
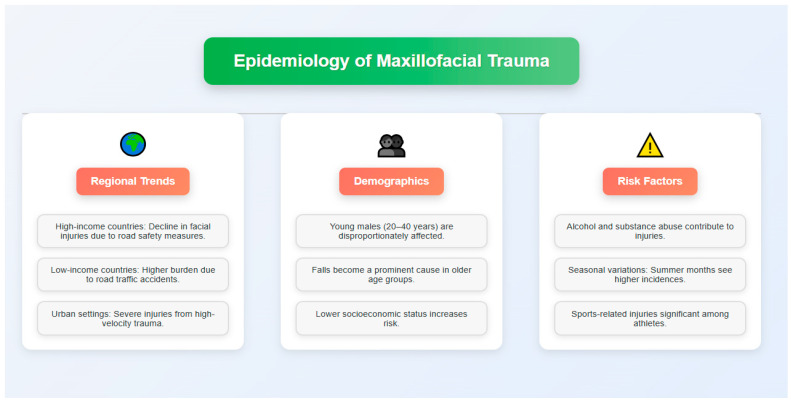
Epidemiology of maxillofacial trauma.

Maxillofacial trauma shows significant regional and demographic variations influenced by socioeconomic, cultural, and environmental factors [24]. While precise global rates are difficult to determine, evidence suggests that 33–50% of trauma cases involve maxillofacial injuries, representing approximately 15% of all emergency department visits worldwide [25]. The geographical distribution of maxillofacial injuries reveals distinct patterns between high-income countries and low/middle-income regions. While high-income countries have experienced decreasing incidence of severe facial trauma due to improved road safety regulations [26], low- and middle-income countries continue to bear a disproportionate burden, primarily due to RTAs, inadequate infrastructure, and limited enforcement of safety regulations [27]. Demographically, young adult males (20–40 years) constitute the highest risk group for maxillofacial trauma, particularly from RTAs and interpersonal violence, reflecting greater risk-taking behavior and occupational exposure [28]. This gender disparity diminishes in older populations, where falls become the predominant cause [29]. Socioeconomic status significantly influences injury risk, with disadvantaged populations experiencing higher rates due to hazardous living/working conditions, limited access to protective equipment, and greater exposure to interpersonal violence [3]. Alcohol and substance abuse serve as major contributing factors across socioeconomic groups [30]. The nature and severity of injuries vary by setting, with urban environments typically presenting more severe, high-velocity trauma from vehicular accidents compared to the lower-velocity injuries common in rural areas [31] (Figure 1). Seasonal variations show increased incidence during summer months and holidays, with Erdmann et al. reporting a 28% higher incidence of maxillofacial fractures during June–August compared to winter months, correlating with increased outdoor activities and higher alcohol consumption [32].

Specific contexts produce distinct injury patterns. Sports-related maxillofacial trauma predominantly affects younger athletes, with Mourouzis and Koumoura documenting that contact sports (boxing, rugby, hockey) accounted for 77% of sports-related facial fractures in their study of 125 patients, with nasal and zygomatic complex fractures being the most common injuries [33]. Occupational injuries occur primarily in construction, manufacturing, and agriculture, with Ramli et al. reporting that workplace accidents constituted 19.3% of maxillofacial trauma cases in their retrospective analysis, though improved safety protocols have reduced their incidence in many regions [34]. During wars and natural disasters, the proportion of high-severity penetrating injuries rises dramatically, with Breeze et al. documenting that 76% of military maxillofacial injuries in Iraq and Afghanistan involved complex ballistic trauma requiring multidisciplinary care, complicated by limited healthcare resources [35]. Recent global events like the COVID-19 pandemic have altered traditional epidemiological patterns, with Salzano et al. reporting a 69.2% decrease in RTA and sports-related injuries during lockdowns counterbalanced by a 58.3% increase in domestic violence-related facial trauma in their analysis of 712 patients [36]. Understanding these epidemiological patterns is essential for developing targeted preventive strategies, appropriate resource allocation, and specialized maxillofacial trauma care services [37].

## 4. Etiology

Maxillofacial trauma etiology varies significantly across geographical regions, reflecting socioeconomic, cultural, and environmental factors. Understanding these causes is essential for developing effective prevention and treatment strategies. RTAs constitute the predominant cause of maxillofacial injuries worldwide, with Mijiti et al. reporting that traffic accidents accounted for 61.5% of maxillofacial fractures in their analysis of 2492 patients, showing particularly high rates in developing countries where the relative risk was 2.7 times higher than in developed nations [4]. Their prevalence correlates with inadequate infrastructure, insufficient traffic regulations, and limited enforcement of safety measures. Urban settings with dense populations and heavy traffic present particularly high risks for severe facial trauma. Interpersonal violence represents another significant etiological factor, with patterns varying across different societies [38]. Contributing elements include alcohol and substance abuse, socioeconomic disparities, and cultural norms. Domestic violence emerges as a particularly challenging subset due to underreporting and intervention difficulties [39]. Falls affect distinct demographic groups, with increasing incidence among elderly populations—often complicated by comorbidities and anticoagulant use [40]. In pediatric patients, falls from heights and playground accidents require specialized management approaches due to the developing facial skeleton [41]. Sports-related maxillofacial injuries, though less common than RTAs, show distinctive patterns with higher rates of isolated fractures and soft tissue damage. Contact sports (boxing, rugby, ice hockey) and extreme sports carry elevated risk profiles [42]. Occupational hazards persist in construction and industrial sectors despite improved workplace safety regulations, with Erdmann et al. reporting that machinery accidents and falling objects accounted for 23.7% of work-related facial injuries in their study of 409 cases, resulting in significantly higher severity scores (mean Facial Injury Severity Scale 3.8 vs. 2.2) compared to other workplace trauma mechanisms [43]. In conflict zones and natural disaster areas, explosions, gunshots, and structural collapses produce complex maxillofacial injury patterns requiring specialized management [44]. Rural communities face unique challenges with animal-related injuries from livestock, wild animals, and domestic pets, with Rahman et al. documenting that animal attacks constituted 8.2% of maxillofacial injuries in rural regions compared to just 1.7% in urban areas, necessitating specific infection control and wound management protocols due to a 3.4-fold higher risk of wound contamination [45]. Alcohol and substance abuse significantly influence trauma patterns by contributing to both RTAs and interpersonal violence. A substantial proportion of maxillofacial trauma patients present with intoxication, with Laverick et al. reporting positive blood alcohol levels in 56% of assault cases and 42% of motor vehicle accidents, and demonstrating that intoxicated patients required hospitalization for 2.4 days longer on average, highlighting the importance of substance abuse prevention within injury reduction strategies [46]. Emerging lifestyle trends introduce new etiological factors, including injuries from personal mobility devices like electric scooters, with Störmann et al. documenting a 184% increase in scooter-related facial fractures between 2019–2021 in their multicenter study, with 72% of patients not wearing helmets [47], and accidents related to distracted walking or driving due to mobile device use. As global patterns evolve with climate change, demographic shifts, and technological advancements, continuous monitoring of etiological trends remains crucial for adapting prevention and treatment approaches to meet changing needs.

## 5. Common Maxillofacial Injuries

Maxillofacial trauma is a broad term that involves injury to the soft tissues, bones, and dentition of the face and associated structures. Due to the intricate anatomy of the facial region along with the importance from both functional and aesthetic perspectives, it is important to be aware of the different varieties of injuries that can happen.

### 5.1. Soft Tissue Injuries

Soft tissue injuries frequently present in maxillofacial trauma, ranging from minor abrasions to extensive lacerations and avulsions, with Hussaini et al. reporting that 78.6% of maxillofacial trauma cases involved soft tissue injuries, with lacerations being the most common (53.2%), followed by contusions (22.9%) and abrasions (15.8%), with 14.5% of cases involving tissue avulsion requiring complex reconstruction [25]. These injuries affect skin, subcutaneous tissues, muscles, and neurovascular structures, with particular concern for scarring and functional impairment. The face’s rich vascularity results in profuse bleeding even from minor wounds, requiring prompt intervention [48]. Blunt trauma typically produces contusions and hematomas, with presentation varying based on impact force and tissue type, with Tadisina et al. demonstrating that high-energy blunt impacts (>50 J) resulted in deep tissue hematomas in 86.3% of cases compared to only 23.7% in low-energy impacts, with associated edema lasting 2.7 times longer in the high-energy group. Injuries to eyelids, lips, and nose warrant special attention due to their functional importance and aesthetic sensitivity, with Chen et al. reporting that periorbital soft tissue injuries required revision surgery at a rate 3.4 times higher than other facial regions, and that nasolabial distortion of more than 2 mm was associated with a 76% patient dissatisfaction rate regardless of otherwise successful repair [49]. Deeper structures like the parotid gland and duct, facial nerve branches, and lacrimal apparatus may also be affected, requiring specialized management.

### 5.2. Facial Bone Fractures

The complexity of facial fractures increases in an upward direction, with injury patterns determined by mechanism, impact force, and intrinsic bone strength [50]. Mandibular fractures represent the most common facial bone injuries, with treatment approaches dictated by their location and pattern. Condylar fractures require particular attention regarding temporal joint function [51]. Midface fractures (Le Fort, zygomaticomaxillary complex, and orbital fractures) present unique diagnostic and management challenges [52], with reconstruction guided by the concept of facial buttresses. Nasal bone fractures, despite their relative “minor” classification, can cause significant functional and aesthetic issues if improperly managed. Their frequency relates to the prominent position and fragility of nasal bones [53]. Frontal sinus fractures, though uncommon, raise serious concerns due to proximity to intracranial structures and potential long-term complications including mucocele formation and meningitis [54].

### 5.3. Dental and Alveolar Injuries

Dental trauma commonly accompanies maxillofacial injuries, ranging from uncomplicated crown fractures to complete tooth avulsion. Treatment aims to restore both immediate function and long-term dental health [55]. The alveolar process, frequently involved in dental injuries, significantly influences tooth stability and prognosis. Various conditions—root fractures, lateral luxation, subluxation, and extrusion—require prompt intervention for optimal outcomes, highlighting the importance of public education regarding emergency dental care [56].

### 5.4. Associated Injuries

Maxillofacial trauma frequently involves related structures, necessitating comprehensive assessment. Traumatic brain injuries (TBI) represent a major concern due to the direct connection between facial and cranial cavities [57]. TBI risk increases proportionally with facial trauma severity, emphasizing the need for neurological evaluation in all facial injury cases. Cervical spine injuries, though less common, require evaluation during initial assessment. The mechanisms causing facial trauma often generate forces that can compromise the cervical spine, warranting appropriate immobilization and evaluation protocols [58]. Ocular trauma ranges from mild contusion to globe rupture, typically requiring interdisciplinary management with ophthalmology consultation. High-energy impacts may overwhelm the orbital bones’ protective function, resulting in direct ocular damage [59]. Airway compromise represents a critical concern in maxillofacial trauma. Research emphasizes airway management as the highest priority, sometimes necessitating advanced interventions including surgical airway establishment [60]. Comprehensive understanding of these injury patterns facilitates effective diagnosis, treatment planning, and multidisciplinary management—balancing functional restoration with aesthetic considerations in these complex cases.

## 6. Economic Impact

The costs of maxillofacial trauma are not limited to acute particular care alone but are extensive in terms of direct and indirect responses to trauma on an economic scale (Table 1). It is important to know the economics of these injuries to formulate prevention strategies, allocate resources availing health care and apply treatment modalities judiciously.

### 6.1. Healthcare Costs

Maxillofacial trauma is estimated to have significant direct healthcare costs due to the complex nature of these injuries and the multidisciplinary approach often required for their management, and costs have an important impact on health system budgeting. A large part of these costs can be attributed to initial emergency department visits, diagnostic imaging, surgical interventions and hospitalization [73].

Costs can range anywhere from simple emergency repair to long-term medical care when multiple facial fractures or associated injuries are involved that require extensive surgical procedures and hospital stays. Advanced imaging techniques, including computed tomography (CT) and magnetic resonance imaging (MRI), although necessary for accurate diagnosis and treatment planning, add considerably to the total cost of care [74].

Additionally, it places a significant financial burden on the patients in need of specialized equipment and materials such as plates, screws, and other fixation devices in maxillofacial surgery. For example, in cases that require urgent surgery, the cost of operating rooms, anesthesia, and post-operative treatment in intensive care settings may significantly impact total spending on healthcare.

Furthermore, treating complications like infections, malunion, or revision surgeries can impose unexpected added costs. Because maxillofacial trauma is more enduring, requiring frequent follow-up visits, imaging studies, and potential secondary procedures, cumulative costs of healthcare tend to increase over time [61].

A further cost for those sustaining extensive dental and alveolar injuries includes dental rehabilitation with prosthetic replacements and implants.

### 6.2. Loss of Productivity

Maxillofacial trauma also has a significant impact on productivity, contributing to this economic burden, particularly as the effects can persist long beyond the initial recovery phase. When patients are hospitalized and need time off work or school during recovery, this is a direct loss of productivity by those individuals, which, in turn, contributes to loss of productivity in the economy as a whole [62].

The duration of work absence can be greatly affected by both the severity and nature of facial injuries. It can be assumed that complex fractures or necessitating extensive surgical reconstruction will lead to prolonged periods of disability with significant loss of income for the affected individuals as well as reduced economic output for the employer.

For example, maxillofacial trauma leading to chronic disfigurement or loss of function can affect long-term employability and work advancement, with Auerbach et al. documenting that patients with moderate to severe facial disfigurement experienced a 34% reduction in job interview success rates and earned on average 22% less than matched controls five years post-injury. This can result in decreased earning capacity throughout the life of the individual, with Morrison et al. calculating that severe maxillofacial trauma resulted in an average lifetime earnings loss of USD 512,000 per patient when accounting for both direct employment effects and missed advancement opportunities, reinforcing the negative economic impact of the initial injury and demonstrating that the economic burden extends far beyond immediate healthcare costs [63]. Even after the physical recovery, the psychological sequelae in the form of post-traumatic stress disorder (PTSD) or depression can also lead to impaired productivity and reduced work performance [64]. These mental health sequelae may require more time off work for treatment and may impact long-term career prospects.

### 6.3. Rehabilitation Expenses

Rehabilitation after maxillofacial trauma is usually long and complex, requiring different types of therapy and interventions, leading to high costs. For temporomandibular joint injuries or facial nerve function related cases, physical therapy may be necessary for longer durations [65].

Often, patients receiving extensive oral or maxillofacial surgery, especially palatal or mandibular reconstruction, require speech therapy. The expense of rehabilitation can vary widely depending on the duration and intensity of these therapies [66].

Psychological support and counseling are another important part of rehabilitation, as facial injuries can take a severe emotional and psychological toll. The prices for mental health services on a long basis can escalate, especially in cases of extreme disfigured or traumatic events [67].

Prosthetic rehabilitation, which can involve facial prostheses for patients with wide loss of tissue, dental implants, and other restorative procedures, represents a large share of rehabilitation costs. Although necessary to restore function or aesthetics, these interventions are often associated with significant material and professional costs [68].

Long-term rehabilitation costs are compounded by the need for ongoing medical management, such as pain management, scar revision techniques and treatment of chronic conditions stemming from the original trauma. Such costs potentially span many years or even a lifetime [69].

## 7. Prevention Strategies

**Figure 2:**
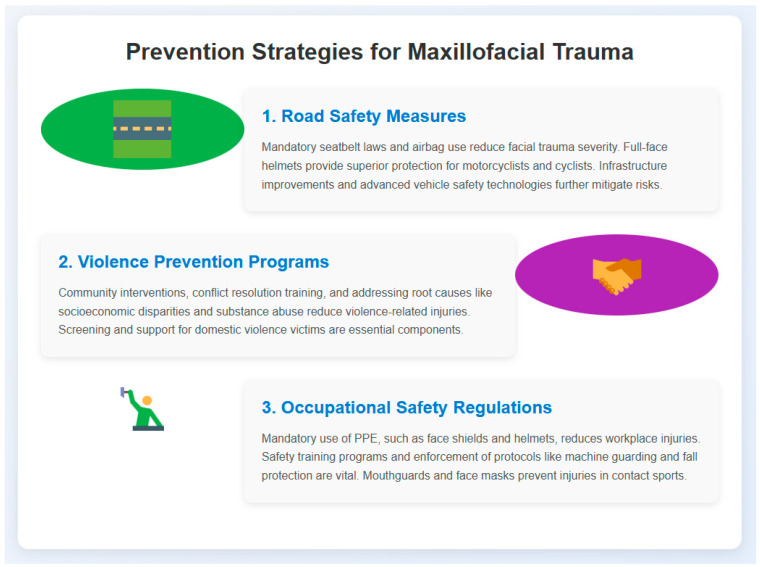
Prevention approach and workplace regulation.

The effective prevention of maxillofacial trauma requires a multifaceted approach addressing various injury mechanisms. The significant personal, economic, and societal burdens associated with facial injuries necessitate collaborative prevention strategies involving policymakers, healthcare professionals, law enforcement, and the public (Figure 2).

### 7.1. Road Safety Measures

RTAs represent the leading cause of maxillofacial injuries globally. Primary seat belt laws have demonstrated significant reductions in facial injury incidence and severity during vehicle crashes (Figure 2). Similarly, airbag technology has reduced overall facial trauma severity in high-impact collisions, despite occasionally causing minor midface injuries [70]. For motorcyclists and cyclists, full-face helmets provide superior protection against facial fractures and soft tissue injuries compared to open-face designs [71]. Jurisdictions implementing mandatory helmet laws combined with educational campaigns have achieved substantial compliance rates and corresponding reductions in facial trauma [72]. Infrastructure improvements, including traffic calming measures and pedestrian–vehicle separation, further reduce accident rates involving vulnerable road users.

### 7.2. Violence Prevention Programs

Interpersonal violence contributes significantly to maxillofacial trauma, necessitating comprehensive prevention programs. Community-based interventions targeting high-risk groups have proven effective in reducing assault-related facial injuries, with Warburton and Shepherd demonstrating a 42% reduction in facial fractures following implementation of their violence prevention program across 32 urban communities, which included targeted education for young males (ages 18–24), alcohol management strategies in high-risk venues, and coordination between law enforcement and local businesses, resulting in annual healthcare savings estimated at USD 1.8 million across the study population [75]. Successful programs incorporate conflict resolution training, anger management, and mentorship (Figure 2).

Addressing underlying factors—social inequality, substance abuse, and mental health issues—requires integrated approaches involving law enforcement, social services, and healthcare providers [76]. Domestic violence, frequently resulting in facial trauma, demands dedicated screening protocols, victim support services, legal protections, and perpetrator rehabilitation programs [77].

### 7.3. Occupational Safety Regulations

Work-related maxillofacial trauma, common in construction, manufacturing, and agriculture, requires robust occupational safety measures. Mandatory personal protective equipment (PPE), including face shields and helmets, has demonstrably reduced facial injury incidence in high-risk environments (Figure 2) [78]. Regular safety training for equipment handling and hazard identification provides essential prevention foundations. Additional measures like machine guarding and fall protection systems effectively limit industrial facial injuries [79]. In sports settings, protective equipment—particularly mouthguards and face masks in contact sports—significantly reduces dental and facial injuries, with Knapik et al. documenting an 82.5% reduction in orofacial trauma among athletes using custom-fitted mouthguards compared to non-users across 12 different contact sports, and Farrington et al. reporting that implementation of mandatory face masks in youth ice hockey reduced facial fractures by 68% and dental injuries by 72% over a 5-year period, demonstrating a clear cost–benefit ratio of 1:7.3 when comparing equipment costs to avoided healthcare expenses [80]. Rule enforcement against dangerous play and athlete education regarding injury risks complement these protective measures.

### 7.4. Awareness Raising Campaigns

Public education plays a crucial role in maxillofacial trauma prevention. Targeted awareness campaigns addressing specific risk factors and populations effectively modify behavior when delivered through diverse media channels, including social media [81]. Educational initiatives highlighting consequences of dangerous behaviors (drunk driving, violence) promote safer choices, while campaigns promoting protective equipment increase compliance in sports and recreational activities [82]. Public awareness regarding initial trauma management improves outcomes. For example, education about proper tooth preservation following avulsion significantly enhances successful reimplantation rates [59]. For pediatric safety, parent education about household hazards, childproofing, and proper car seat installation reduces facial injuries in children, with Thompson et al. reporting that comprehensive parental education programs resulted in a 47% reduction in home-based facial trauma among children under age 5, and Durbin et al. demonstrating that correctly installed car seats reduced maxillofacial injuries in vehicle crashes by 74% compared to improperly installed restraints, with their longitudinal study of 3216 families showing that intervention groups maintained 83% compliance with safety recommendations at 24-month follow-up versus 36% in control groups [83]. Successful implementation requires cross-sector collaboration. While legislation establishes the framework through regulations against drunk driving and mandatory protective equipment, effectiveness ultimately depends on public compliance and community engagement. Ongoing research examining injury patterns, risk factors, and intervention efficacy remains essential for adapting strategies to evolving conditions, thereby reducing the burden of maxillofacial injuries and enhancing public health outcomes.

## 8. Surgical Management

Surgical intervention for maxillofacial trauma is a dynamic adaptable field that demands a thorough knowledge of facial anatomy, biomechanics, and aesthetic principles. The aims of surgical intervention are threefold: to improve and/or preserve function, to prevent complications, and to maximize long-term outcomes. Surgical management varies based on injury type and severity, as well as patient factors.

### 8.1. Timing of Interventions

Intervention timing significantly influences treatment outcomes. For isolated facial fractures without substantial soft tissue compromise, early definitive management within 72 h of injury is preferred [84], preventing reduction and fixation complications associated with developing edema and fibrosis. For patients with multiple traumas or those requiring critical care, a staged approach may be necessary. Damage control principles, initially developed for abdominal trauma, have been successfully adapted to maxillofacial injuries [33]. This involves initial stabilization of life-threatening conditions followed by definitive reconstruction once the patient’s overall condition stabilizes. Complex panfacial fractures require systematic surgical sequencing, typically beginning with mandibular arch restoration [85]. This establishes proper occlusion and facial width, creating a foundation for subsequent reconstruction.

### 8.2. Surgical Approaches

Approach selection balances adequate exposure for fracture reduction and fixation with minimizing aesthetic and functional impairment. Contemporary techniques favor minimally invasive approaches utilizing pre-existing scars or natural skin lines whenever possible [86]. Intraoral approaches predominate for mandibular fractures to avoid visible scarring, though external approaches may be necessary for complex or comminuted fractures, particularly involving the condyle or ramus [87]. Endoscopic-assisted techniques have gained popularity for subcondylar fractures, offering enhanced visualization with minimal external incisions [88]. For upper and middle facial thirds, the coronal approach provides versatile access, though less invasive alternatives such as transconjunctival approaches for orbital floor fractures and sublabial approaches for maxillary fractures have gained favor for their reduced morbidity and improved aesthetic outcomes [89].

### 8.3. Reconstruction Techniques

Modern reconstruction addresses both bony and soft tissue defects, often through combined techniques. Fixation methods follow load-sharing and load-bearing osteo-synthesis principles [90], with advancements in plating systems and resorbable materials expanding surgical options. Advanced technologies have revolutionized maxillofacial reconstruction, with virtual surgical planning (VSP) emerging as a cornerstone approach. VSP enables surgeons to simulate complex procedures virtually before surgery, significantly reducing operating time and improving surgical precision [91]. The workflow typically begins with CT data acquisition, followed by digital segmentation, virtual manipulation of bony segments, and creation of surgical guides or implants [92]. Studies demonstrate that VSP reduces operating time by 30–45% in complex reconstructions while improving symmetry outcomes [93]. Patient-specific implants (PSIs) have transformed complex orbital fracture management. These custom implants, designed via computer-aided design and manufacturing (CAD/CAM), effectively restore orbital volume and contour with unprecedented precision [94]. A retrospective analysis of 124 orbital reconstructions revealed that PSIs reduced reoperation rates from 18% to 3.2% compared to traditional techniques [95]. Similarly, mandibular reconstruction accuracy has significantly improved through VSP and three-dimensional printing technologies. Fibula free flaps designed using VSP demonstrate mean deviation of only 1.2 mm from planned outcomes compared to 3.8 mm using conventional techniques. These technologies enable precise osteotomy planning, accurate bony segment positioning, and creation of cutting guides that facilitate intraoperative execution of the virtual plan. Soft tissue reconstruction ranges from local and regional flaps for smaller defects to microvascular free tissue transfer for extensive tissue loss. Flap design follows facial subunit principles to optimize aesthetic outcomes [96]. Computer-assisted flap planning has improved tissue volume calculations and recipient site matching, particularly for complex cases requiring chimeric flaps [97]. Refined fat grafting techniques provide valuable tools for volume restoration and contour refinement during later reconstructive stages [98]. Panfacial fracture management demands meticulous restoration of facial buttress anatomy, typically sequenced from the outer facial frame (mandible and zygoma) to central midface structures [99]. Computer-assisted navigation systems have substantially improved reduction and fixation accuracy in these complex cases, with real-time feedback reducing positional errors to less than 1 mm in 94% of cases [100]. Intraoperative navigation particularly benefits complex zygomatic–orbital–maxillary reconstructions where subtle malposition can result in significant functional and aesthetic compromise. For catastrophic tissue deficits where primary reconstruction proves inadequate, facial transplantation represents a pioneering solution. Though still experimental, this procedure offers unprecedented restoration of both form and function in select patients with devastating facial trauma [101]. Recent advances in immunosuppressive protocols have improved long-term outcomes, with five-year graft survival rates improving from 50% to 80% in the past decade [102]. Emerging technologies are increasingly integrated into surgical workflows, enhancing precision and outcome predictability. Augmented reality (AR) systems allow surgeons to visualize underlying bony structures during surgery, improving accuracy while reducing invasiveness [103]. Novel bioactive materials like calcium phosphate-based scaffolds with growth factor delivery systems show promising results in accelerating bone regeneration in critical-sized defects [104]. Advances in tissue engineering and regenerative medicine promise further improvements in soft tissue reconstruction and bone regeneration capabilities through development of cell-seeded constructs that promote tissue integration and vascularization [105].

## 9. Complications and Long-Term Outcomes

Maxillofacial trauma is a heterogeneous entity, and although, with improvements in surgical techniques and perioperative management, complication rates have dropped, these still exist. Complications can be immediate and postoperative, but also structural in terms of long-standing gagging functional and psychological sequelae ranging. Early recognition of and anticipation of possible complications is paramount for best patient care and optimizing outcomes.

### 9.1. Immediate Complications

Immediate complications following maxillofacial trauma encompass infectious, vascular, and neurological issues. Infection remains a significant concern, particularly in compound fractures with compromised soft tissue coverage. Postoperative infection rates range from 3% to 33%, varying with injury severity and treatment approach [99,100,101,102]. The most common microorganisms isolated from maxillofacial infections include staphylococcus aureus (42%), streptococcus species (28%), anaerobes such as Peptostreptococcus and Bacteroides (18%), and Gram-negative bacteria including Klebsiella and Pseudomonas (12%) [103]. This microbial profile guides empirical antibiotic therapy, with first-line treatment typically including broad-spectrum coverage with amoxicillin-clavulanate or clindamycin for penicillin-allergic patients. For contaminated wounds, particularly those involving soil or foreign bodies, tetanus prophylaxis with tetanus toxoid and, in high-risk cases, tetanus immunoglobulin is essential within 24 h of injury [104]. These risks can be mitigated through proper wound care, appropriate antibiotic prophylaxis, and precise surgical technique. Vascular complications, though less common, present serious risks including hematoma formation with potential airway compromise, particularly in the neck and floor of mouth [100]. Vigilant postoperative monitoring and rapid intervention are crucial for managing these complications. Rare but serious complications include pseudoaneurysms of the facial artery or external carotid branches, requiring endovascular or surgical management [101]. In addition to localized complications, maxillofacial trauma can lead to more serious intracranial sequelae. Cerebral edema may develop in cases with concomitant head injury or when maxillofacial fractures extend into the cranial base, requiring urgent neurosurgical consultation and aggressive management with osmotic diuretics and careful intracranial pressure monitoring [105]. Infections from the maxillofacial region can spread to the cavernous sinus, resulting in cavernous sinus thrombosis—a rare but potentially life-threatening complication characterized by ophthalmoplegia, chemosis, and rapidly progressive systemic symptoms requiring immediate administration of high-dose intravenous antibiotics and possibly anticoagulation [106]. Neurological complications primarily involve cranial nerve damage, with facial nerve injury occurring in 7–10% of temporal bone fractures [102]. Early identification and appropriate intervention, including surgical exploration and potential nerve repair, significantly improve outcomes. Immediate complications following maxillofacial trauma encompass infectious, vascular, and neurological issues. Infection remains a significant concern, particularly in compound fractures with compromised soft tissue coverage. Postoperative infection rates range from 3% to 33%, varying with injury severity and treatment approach [103]. These risks can be mitigated through proper wound care, appropriate antibiotic prophylaxis, and precise surgical technique. Vascular complications, though less common, present serious risks including hematoma formation with potential airway compromise, particularly in the neck and floor of mouth [100]. Vigilant postoperative monitoring and rapid intervention are crucial for managing these complications. Rare but serious complications include pseudoaneurysms of the facial artery or external carotid branches, requiring endovascular or surgical management [101]. Neurological complications primarily involve cranial nerve damage, with facial nerve injury occurring in 7–10% of temporal bone fractures [102]. Early identification and appropriate intervention, including surgical exploration and potential nerve repair, significantly improve outcomes.

### 9.2. Long-Term Functional Disability

Functional disabilities following maxillofacial trauma substantially impact quality of life. Malocclusion represents one of the most common sequelae after mandibular or maxillary fractures, with reported incidence ranging from 3% to 25% depending on fracture type and treatment [103]. Severe cases unresponsive to orthodontic treatment may necessitate secondary orthognathic surgery. Temporomandibular joint dysfunction frequently follows condylar fractures, with up to 30% of patients experiencing symptoms including pain, limited mouth opening, and joint sounds [104]. Management typically begins with conservative approaches (physical therapy, occlusal splints) before considering surgical intervention for refractory cases. Visual impairment may result from orbital fractures or associated optic nerve injuries. Despite improved surgical techniques reducing post-traumatic enophthalmos and diplopia incidence, these remain significant concerns in complex orbital fractures [105], often requiring regular ophthalmologic follow-up and potential corrective surgery. Nasal airway obstruction frequently complicates nasal and nasoethmoid complex fractures, affecting approximately 40% of patients [106]. Secondary septorhinoplasty may be required to address both functional and aesthetic concerns.

### 9.3. Psychological Impact

The psychological burden of maxillofacial trauma represents an often-underestimated aspect of patient management. The visible nature of facial injuries significantly impacts body image, self-esteem, and social interactions. PTSD affects up to 27% of facial trauma patients within the first year post-injury [65], with functional impairment and facial disfigurement associated with more severe symptoms. Depression and anxiety are also common, with prevalence rates between 20% and 40% [67]. These psychological effects extend beyond patients to their families and caregivers, with relatives of severely injured patients experiencing elevated emotional distress requiring support and counseling [107]. The early identification of psychologically vulnerable patients is crucial. Screening tools such as the Hospital Anxiety and Depression Scale (HADS) and Impact of Event Scale-Revised (IES-R) help identify patients who would benefit from psychological intervention [108]. Cognitive-behavioral therapy has demonstrated effectiveness in treating PTSD and depression in facial trauma patients [109], while support groups and peer counseling provide valuable emotional support and coping strategies. In maxillofacial trauma management, aesthetic outcomes significantly influence social interaction and self-image. While functional restoration remains the primary objective, patients’ psychological recovery and treatment satisfaction depend substantially on aesthetic results [108], highlighting the importance of addressing both functional and aesthetic concerns in comprehensive treatment planning.

## 10. Conclusions

Maxillofacial trauma remains a significant global health challenge, with far-reaching physical, psychological, and socioeconomic consequences. Addressing this issue requires a multifaceted approach that encompasses improved prevention strategies, enhanced access to specialized care, and the development of innovative treatment modalities.

Future directions in maxillofacial trauma management should focus on leveraging advanced technologies, such as 3D printing and virtual surgical planning, to optimize surgical outcomes and reduce recovery times. Additionally, efforts should be made to bridge the global disparities in care through international collaborations, telemedicine initiatives, and targeted training programs for healthcare providers in resource-limited settings.

Ultimately, a comprehensive and coordinated approach involving healthcare professionals, policymakers, and community stakeholders will be essential to reduce the incidence and impact of maxillofacial trauma worldwide.

## Figures and Tables

**Table 1:** Maxillofacial trauma and economic impact and ICU.

Economic Factor	Description	Key Findings	Implementation Challenges	Affected Groups	References
Direct Healthcare Costs	Immediate medical expenses for treatment of facial injuries	Mean hospital charges of USD 55,385 per admission; annual cost of USD 1.06 billion in the US	High variability in costs across regions and facilities	Patients, hospitals, insurance providers	[45]
Surgical Intervention Costs	Costs for surgical procedures, including reconstruction and fixation	Severe cases may cost up to USD 200,000 per patient	Limited access to advanced surgical facilities in low-income regions	Patients with severe injuries	[61,62,63,64]
Imaging and Diagnostic Costs	Expenses for CT, MRI, and other imaging required for diagnosis and surgical planning	Imaging is essential but significantly contributes to overall costs	Limited availability of CT/MRI in resource-poor settings	Trauma patients, healthcare facilities	[63]
Long-term Follow-up Costs	Expenses for follow-up visits and secondary procedures	Recovery often requires multiple follow-ups, adding to cumulative healthcare expenditure	Compliance with follow-up care can be challenging	Patients requiring ongoing care	[64]
Loss of Productivity	Time away from work or school due to hospitalization and recovery	An average of 11.8 days lost per incident; substantial economic loss in affected regions	Long recovery periods or permanent disability	Workers, employers, families	[65]
Long-term Employment Impact	Effects on career progression and earning capacity	Up to 30% of severely injured patients face long-term unemployment or underemployment	Limited opportunities for rehabilitation and retraining	Low-income workers, severely injured	[66]
Rehabilitation Expenses	Costs for therapies (physical, speech, psychological), prosthetics, and long-term care	Patients require an average of 22 therapy sessions over six months, costing ~USD 3800 per patient	Access to therapy facilities and affordability issues	Trauma patients, rehab centers	[67,68]
Dental Rehabilitation Costs	Costs for dental implants, prosthetics, and restorations	Average cost of USD 18,000 per patient; complex cases can exceed USD 50,000	Insurance may not cover dental implants; affordability for patients	Patients with dental injuries	[69]
Psychological Treatment Costs	Mental health care costs for PTSD, depression, and anxiety associated with disfigurement	Often underestimated but essential for long-term recovery	Stigma around mental health; availability of specialized care	Patients, mental health providers	[70,71]
Impact on Uninsured Patients	Financial burden on individuals without insurance coverage	22.4% of patients with facial fractures are uninsured, leading to increased out-of-pocket expenses	Limited access to affordable care options	Uninsured and underinsured populations	[72]

CT: Computed Tomography, MRI: Magnetic Resonance Imaging, US: United States, PTSD: Post-Traumatic Stress Disorder.

## References

[1] Park H., Oh S., Ra Y.-S. (2022). Worldwide epidemiology of maxillofacial fractures: A systematic review and meta-analysis. J. Craniomaxillofac Surg..

[2] Patil S.G., Patil B.S., Joshi U. (2022). The Contemporary Management of Maxillofacial Trauma: A Review. J. Maxillofac. Oral. Surg..

[3] Boffano P., Roccia F., Zavattero E., Dediol E., Uglešić V., Kovačič Ž., Vesnaver A., Konstantinović V.S., Petrović M., Stephens J. (2015). European Maxillofacial Trauma (EURMAT) project: A multicentre and prospective study. J. Craniomaxillofac Surg..

[4] Singaram M., Udhayakumar R.K. (2016). Prevalence, pattern, etiology, and management of maxillofacial trauma in a developing country: A retrospective study. J. Korean Assoc. Oral Maxillofac. Surg..

[5] Alkhabuli J., Alnaqbi H., Aisha A. (2020). Etiology and pattern of maxillofacial fractures: A 10-year retrospective analysis of cases in Ras Al-Khaimah, United Arab Emirates. Oral. Health Dent. Manag..

[6] Zargar M.R., Khaji A., Karbakhsh M., Reza Zarei M. (2016). Epidemiology study of facial injuries during a 13 month of trauma registry in Tehran. Indian. J. Med. Sci..

[7] Katsaros T., Balasubramanian S., Basyuni S. (2021). The epidemiology of facial trauma in major trauma patients. Surgeon.

[8] Tuckett J., Lynham A., Lee G., Perry M., Harrington U. (2018). Maxillofacial trauma in the emergency department: A review. Surgeon.

[9] Choi S.H., Cha J.Y., Kang D.Y. (2021). Precision and accuracy of automated 3D cephalometric landmark identification with CNN-based segmentation method. Sci. Rep..

[10] Murphy C., Oguz I., Shetye P.R. (2021). Machine learning-based prediction of post-trauma facial growth in children with facial fractures. Laryngoscope.

[11] Girotto J.A., MacKenzie E., Fowler C., Redett R., Robertson B., Manson P.N. (2001). Long-term physical impairment and functional outcomes after complex facial fractures. Plast. Reconstr. Surg..

[12] Kaura A., Leitner L., Grant C. (2020). Psychological impact of facial trauma: A systematic review. Br. J. Oral. Maxillofac. Surg..

[13] Zaid M., Shetty V., Glynn S. (2021). Psychosocial factors in trauma recovery: A systematic review. Dent. Traumatol..

[14] Van Boven G., Dijkema T., Raghoebar G.M. (2023). Global inequality in access to oral and maxillofacial surgical care. Lancet Glob. Health.

[15] World Health Organization (2023). Global Status Report on Road Safety 2023.

[16] World Health Organization (2019). World Report on Violence and Trauma Prevention.

[17] Shumrick K.A., Campbell A.C., Timashpolsky A. (2018). Advances in radiographic evaluation of facial trauma. J. Craniofac Surg..

[18] Metzler P., Geiger E.J., Alcon A. (2020). Three-dimensional virtual surgical planning for patient-specific oromandibular reconstruction using a segmented fibula. J. Craniofac Surg..

[19] Ahn Y.S., Kim S.G., Baik S.M., Kim B.O., Kim H.K., Moon S.Y. (2018). Comparative study between resorbable and nonresorbable plates in orthognathic surgery. J. Oral. Maxillofac. Surg..

[20] Meara J.G., Leather A.J., Hagander L., Alkire B.C., Alonso N., Ameh E.A., Bickler S.W., Conteh L., Dare A.J., Davies J. (2015). Global Surgery 2030: Evidence and solutions for achieving health, welfare, and economic development. Lancet.

[21] Alkire B.C., Raykar N.P., Shrime M.G. (2023). Global access to surgical care: A modelling study. Lancet Glob. Health.

[22] Chauhan V., Khan M.A., Raval C.B. (2022). Telemedicine for maxillofacial trauma triage during the COVID-19 pandemic and beyond: A systematic review. J. Stomatol. Oral. Maxillofac. Surg..

[23] Olsson P., Svensson S., Johansen K. (2022). A systematic review of the etiology and pattern of maxillofacial fractures in elderly patients. Int. J. Oral. Maxillofac. Surg..

[24] Chrcanovic B.R. (2012). Factors influencing the incidence of maxillofacial fractures. Oral. Maxillofac. Surg..

[25] Gassner R., Tuli T., Hächl O., Rudisch A., Ulmer H. (2003). Cranio-maxillofacial trauma: A 10 year review of 9,543 cases with 21,067 injuries. J. Craniomaxillofac Surg..

[26] Tamimi I., Alabdullah K., Alshaalan H. (2022). Interpersonal violence-related facial injuries in Saudi Arabia: A nationwide analysis. J. Craniomaxillofac Surg..

[27] Naddumba E.K. (2004). A cross sectional retrospective study of boda boda injuries at Mulago Hospital in Kampala, Uganda. East. Cent. Afr. J. Surg..

[28] Schnitzer M.G., Evangelista F.C., dos Santos B.C. (2023). Mandibular fractures: A 10-year retrospective epidemiological study in Brazil. J. Oral. Maxillofac. Surg. Med. Pathol..

[29] Iida S., Kogo M., Sugiura T., Mima T., Matsuya T. (2001). Retrospective analysis of 1502 patients with facial fractures. Int. J. Oral. Maxillofac. Surg..

[30] Lee K.H. (2009). Interpersonal violence and facial fractures. J. Oral. Maxillofac. Surg..

[31] Sane V.D., Rathi S.S., Kondekar A. (2022). Changing patterns of maxillofacial trauma in India during COVID-19 pandemic: A comparative retrospective study. J. Maxillofac. Oral. Surg..

[32] Erdmann D., Follmar K.E., Debruijn M., Bruno A.D., Jung S.H., Edelman D., Mukundan S., Marcus J.R. (2008). A retrospective analysis of facial fracture etiologies. Ann. Plast. Surg..

[33] Mourouzis C., Koumoura F. (2005). Sports-related maxillofacial fractures: A retrospective study of 125 patients. Int. J. Oral. Maxillofac. Surg..

[34] Ramli R., Rahman N.A., Rahman R.A., Hussaini H.M., Hamid A.L.A. (2011). A retrospective study of oral and maxillofacial injuries in Seremban Hospital, Malaysia. Dent. Traumatol..

[35] Breeze J., Gibbons A.J., Hunt N.C., Monaghan A.M., Gibb I., Hepper A. (2011). Mandibular fractures in British military personnel secondary to blast trauma sustained in Iraq and Afghanistan. Br. J. Oral. Maxillofac. Surg..

[36] Salzano G., Dell’Aversana Orabona G., Audino G., Vaira L.A., Trevisiol L., D’Agostino A. (2021). Have there been any changes in the epidemiology and etiology of maxillofacial trauma during the Italian lockdown for the COVID-19 epidemic? An analysis of 712 injuries received during the pandemic. J. Craniomaxillofac Surg..

[37] Boffano P., Kommers S.C., Karagozoglu K.H., Forouzanfar T. (2014). Aetiology of maxillofacial fractures: A review of published studies during the last 30 years. Br. J. Oral. Maxillofac. Surg..

[38] Lee K. (2012). Global trends in maxillofacial fractures. Craniomaxillofac Trauma. Reconstr..

[39] Wong J.Y., Choi A.W., Fong D.Y., Wong J.K., Lau C.L., Kam C.W. (2014). Patterns, aetiology and risk factors of intimate partner violence-related injuries to head, neck and face in Chinese women. BMC Womens Health.

[40] Yamamoto K., Kuraki M., Kurihara M., Matsusue Y., Murakami K., Horita S., Sugiura T., Kirita T. (2010). Maxillofacial fractures resulting from falls. J. Oral. Maxillofac. Surg..

[41] Lee K.S., Lee J.H., Kim S.M. (2020). Management of maxillofacial trauma in pediatric patients: A nationwide multicenter study in South Korea. J. Craniofac Surg..

[42] Vieira R.C.A., de Melo G.P., Antunes A.A., Dourado E., de Barros Silva P.G. (2014). The influence of extreme sports in the prevalence of maxillofacial fractures: A retrospective study of 72 cases. Oral. Maxillofac. Surg..

[43] Roccia F., Bianchi F., Zavattero E., Tanteri G., Ramieri G. (2010). Characteristics of maxillofacial trauma in females: A retrospective analysis of 367 patients. J. Craniomaxillofac Surg..

[44] Breeze J., Gibbons A.J., Shieff C., Banfield G., Bryant D.G., Midwinter M.J. (2011). Combat-related craniofacial and cervical injuries: A 5-year review from the British military. J. Trauma. Acute Care Surg..

[45] Ugboko V.I., Olasoji H.O., Ajike S.O., Amole A.O.D., Ogundipe O.T. (2002). Facial injuries caused by animals in northern Nigeria. Br. J. Oral. Maxillofac. Surg..

[46] O’Meara C., Witherspoon R., Hapangama N., Hyam D.M. (2012). Alcohol and interpersonal violence may increase the severity of facial fracture. Br. J. Oral. Maxillofac. Surg..

[47] Namiri N.K., Lui H., Tangney T., Allen I.E., Cohen A.J., Breyer B.N. (2020). Electric scooter injuries and hospital admissions in the United States, 2014–2018. JAMA Surg..

[48] Perry M., Holmes S. (2014). Atlas of Operative Maxillofacial Trauma Surgery: Primary Repair of Facial Injuries.

[49] Hollier L.H., Sharabi S.E., Koshy J.C., Stal S. (2010). Facial trauma: General principles of management. J. Craniofac Surg..

[50] Alvi A., Doherty T., Lewen G. (2003). Facial fractures and concomitant injuries in trauma patients. Laryngoscope.

[51] Biçakci A.A., Büyüksoylu O., Yilmaz Y. (2022). Current trends in treatment of mandibular condyle fractures: A systematic review and meta-analysis. J. Stomatol. Oral. Maxillofac. Surg..

[52] Abdelghani M., Durand P., Nasser M. (2021). Reduction and fixation sequences in panfacial fractures: A systematic review and meta-analysis. J. Craniomaxillofac Surg..

[53] Hwang K., You S.H., Kim S.G. (2007). Analysis of nasal bone fractures; a six-year study of 503 patients. J. Craniofac Surg..

[54] Metzinger S.E., Guerra A.B., Garcia R.E. (2005). Frontal sinus fractures: Management guidelines. Facial Plast. Surg..

[55] Andreasen J.O., Andreasen F.M., Andersson L. (2018). Textbook and Color Atlas of Traumatic Injuries to the Teeth.

[56] Andersson L., Andreasen J.O., Day P., Heithersay G., Trope M., DiAngelis A.J., Kenny D.J., Sigurdsson A., Bourguignon C., Flores M.T. (2012). International Association of Dental Traumatology guidelines for the management of traumatic dental injuries: 2. Avulsion of permanent teeth. Dent. Traumatol..

[57] Rajandram R.K., Syed Omar S.N., Rashdi M.F.N., Abdul Jabar M.N. (2014). Maxillofacial injuries and traumatic brain injury—A pilot study. Dent. Traumatol..

[58] Mulligan R.P., Friedman J.A., Mahabir R.C. (2010). A nationwide review of the associations among cervical spine injuries, head injuries, and facial fractures. J. Trauma. Acute Care Surg..

[59] Al-Qurainy I.A., Stassen L.F., Dutton G.N., Moos K.F., El-Attar A. (1991). The characteristics of midfacial fractures and the association with ocular injury: A prospective study. Br. J. Oral. Maxillofac. Surg..

[60] Perry M., Morris C. (2008). Advanced trauma life support (ATLS) and facial trauma: Can one size fit all? Part 2: ATLS, maxillofacial injuries and airway management dilemmas. Int. J. Oral. Maxillofac. Surg..

[61] Krug E.G., Sharma G.K., Lozano R. (2000). The global burden of injuries. Am. J. Public. Health.

[62] Glynn S.M., Shetty V., Elliot-Brown K., Leathers R., Belin T.R., Wang J. (2007). Chronic posttraumatic stress disorder after facial injury: A 1-year prospective cohort study. J. Trauma. Acute Care Surg..

[63] Potter J.K., Asteriadis S., Likavec M.J. (2005). Middle and upper facial fractures: A retrospective review of 234 patients. J. Oral. Maxillofac. Surg..

[64] Levine E., Degutis L., Pruzinsky T., Shin J., Persing J.A. (2005). Quality of life and facial trauma: Psychological and body image effects. Ann. Plast. Surg..

[65] Fonseca R.J., Walker R.V., Betts N.J., Barber H.D., Powers M.P. (2013). Oral and Maxillofacial Trauma.

[66] Scherer M., Sullivan W.G., Smith D.J., Phillips L.G., Robson M.C. (1989). An analysis of 1423 facial fractures in 788 patients at an urban trauma center. J. Trauma. Acute Care Surg..

[67] Islam S., Ahmed M., Walton G.M., Dinan T.G., Hoffman G.R. (2010). The association between depression and anxiety disorders following facial trauma--a comparative study. Injury.

[68] Brennan P.A., Schliephake H., Ghali G.E., Cascarini L. (2017). Maxillofacial Surgery.

[69] Peled M., Leiser Y., Emodi O., Krausz A. (2012). Treatment protocol for high velocity/high energy gunshot injuries to the face. Craniomaxillofac Trauma. Reconstr..

[70] Singleton M., Qin H., Luan J. (2004). Factors associated with higher levels of injury severity in occupants of motor vehicles that were severely damaged in traffic crashes in Kentucky, 2000–2001. Traffic Inj. Prev..

[71] Zhu W., Zhou Y., Jiang F. (2021). The effectiveness of helmet use on reducing motorcycle-related maxillofacial injuries: An updated meta-analysis. Int. J. Environ. Res. Public Health.

[72] Feng S., Li Z., Du Y. (2022). Urban traffic engineering interventions to reduce road traffic injuries: A systematic review and meta-analysis. Lancet Planet. Health.

[73] Allareddy V., Allareddy V., Nalliah R.P. (2011). Epidemiology of facial fracture injuries. J. Oral Maxillofac. Surg..

[74] Deng H., Yang Z., Li J. (2021). Applications of 3D printing technology in the COVID-19 response: A literature review. Front. Med..

[75] Florence C., Shepherd J., Brennan I., Simon T. (2011). Effectiveness of anonymised information sharing and use in health service, police, and local government partnership for preventing violence related injury: Experimental study and time series analysis. BMJ.

[76] Krug E.G., Mercy J.A., Dahlberg L.L., Zwi A.B. (2002). The world report on violence and health. Lancet.

[77] Campbell J.C. (2002). Health consequences of intimate partner violence. Lancet.

[78] Lipscomb H.J. (2000). Effectiveness of interventions to prevent work-related eye injuries. Am. J. Prev. Med..

[79] Lombardi D.A., Verma S.K., Brennan M.J., Perry M.J. (2009). Factors influencing worker use of personal protective eyewear. Accid. Anal. Prev..

[80] Newsome P.R., Tran D.C., Cooke M.S. (2001). The role of the mouthguard in the prevention of sports-related dental injuries: A review. Int. J. Paediatr. Dent..

[81] Wakefield M.A., Loken B., Hornik R.C. (2010). Use of mass media campaigns to change health behaviour. Lancet.

[82] Finch C.F., Donaldson A. (2010). A sports setting matrix for understanding the implementation context for community sport. Br. J. Sports Med..

[83] Durbin D.R., Chen I., Smith R., Elliott M.R., Winston F.K. (2005). Effects of seating position and appropriate restraint use on the risk of injury to children in motor vehicle crashes. Pediatrics.

[84] Ellis E. (2019). Timing of definitive management of facial fractures. J. Oral Maxillofac. Surg..

[85] Manson P.N., Clark N., Robertson B., Slezak S., Wheatly M., Vander Kolk C., Ilif N. (1999). Subunit principles in midface fractures: The importance of sagittal buttresses, soft-tissue reductions, and sequencing treatment of segmental fractures. Plast. Reconstr. Surg..

[86] Chrcanovic B.R. (2012). Open versus closed reduction: Comminuted mandibular fractures. Oral Maxillofac. Surg..

[87] Wei H., Liu Y., Gao Y. (2020). Management of mandibular condylar fractures using intraoral approach: A retrospective analysis of 356 cases. Int. J. Oral Maxillofac. Surg..

[88] Tan Y., Chen X., Hu J. (2021). Endoscopic-assisted management of mandibular condylar fractures: A 10-year review of surgical outcomes. J. Craniomaxillofac Surg..

[89] Salgarelli A.C., Bellini P., Landini B., Multinu A., Consolo U. (2010). A comparative study of different approaches in the treatment of orbital trauma: An experience based on 274 cases. Oral. Maxillofac. Surg..

[90] Khadembaschi D., Smeets R., Jung S. (2023). Biodegradable versus titanium osteosynthesis materials for internal fixation of maxillofacial fractures: A systematic review. Int. J. Oral Maxillofac. Surg..

[91] Gander T., Essig H., Metzler P., Lindhorst D., Dubois L., Rücker M., Schumann P. (2015). Patient specific implants (PSI) in reconstruction of orbital floor and wall fractures. J. Craniomaxillofac Surg..

[92] García-Mato D., Ochandiano S., García-Sevilla M. (2021). Advances in intraoperative navigation and real-time validation in craniomaxillofacial surgery: A systematic review. J. Clin. Med..

[93] Pu L.L.Q., Coleman S.R., Cui X. (2022). Autologous fat grafting in the face: Technical considerations and management of complications. J. Craniofac Surg..

[94] Coleman S.R. (2006). Structural fat grafting: More than a permanent filler. Plast. Reconstr. Surg..

[95] Markowitz B.L., Manson P.N. (1989). Panfacial fractures: Organization of treatment. Clin. Plast. Surg..

[96] Sosin M., Ceradini D.J., Levine J.P., Hazen A., Staffenberg D.A., Saadeh P., Flores R., Sweeney N., Bernstein G., Leslie M. (2016). Total face, eyelids, ears, scalp, and skeletal subunit transplant: A reconstructive solution for the full face and total scalp burn. Plast. Reconstr. Surg..

[97] Mendez B.M., Chiodo M.V., Patel P.A. (2015). Customized “in-office” three-dimensional printing for virtual surgical planning in craniofacial surgery. J. Craniofac. Surg..

[98] Ma J., Both S.K., Yang F., Cui F.-Z., Pan J., Meijer G.J., Jansen J.A., van den Beucken J.J.J.P. (2014). Concise review: Cell-based strategies in bone tissue engineering and regenerative medicine. Stem Cells Transl. Med..

[99] Andreasen J.O., Jensen S.S., Schwartz O., Hillerup Y. (2006). A systematic review of prophylactic antibiotics in the surgical treatment of maxillofacial fractures. J. Oral. Maxillofac. Surg..

[100] Cogbill T.H., Cothren C.C., Ahearn M.K., Cullinane D.C., Kaups K.L., Scalea T.M., Maggio L., Brasel K.J., Harrison P.B., Patel N.Y. (2008). Management of maxillofacial injuries with severe oronasal hemorrhage: A multicenter perspective. J. Trauma. Acute Care Surg..

[101] Bouloux G.F., Perciaccante V.J. (2004). Massive hemorrhage during oral and maxillofacial surgery: Ligation of the external carotid artery or embolization?. J. Oral. Maxillofac. Surg..

[102] Ulug T., Ulubil S.A. (2005). Management of facial paralysis in temporal bone fractures: A prospective study analyzing 11 operated fractures. Am. J. Otolaryngol..

[103] Christensen J., Sawatari Y., Peleg M. (2015). High-energy traumatic maxillofacial injury. J. Craniofac. Surg..

[104] Al-Moraissi E.A., Ellis E. (2014). What method for management of unilateral mandibular angle fractures has the lowest rate of postoperative complications? A systematic review and meta-analysis. J. Oral Maxillofac. Surg..

[105] Kunz C., Sigron G.R., Jaquiéry C. (2013). Functional outcome after non-surgical management of orbital fractures--the bias of decision-making according to size of defect: Critical review of 48 patients. Br. J. Oral Maxillofac. Surg..

[106] Mondin V., Rinaldo A., Ferlito A. (2005). Management of nasal bone fractures. Am. J. Otolaryngol..

[107] Conforte J.J., Alves C.P., Sánchez M.D.P., Ponzoni D. (2016). Impact of trauma and surgical treatment on quality of life of patients with facial fractures. Int. J. Oral Maxillofac. Surg..

[108] Vishwanath K., Prasad R., Thakur D. (2020). Psychological impact of facial trauma: A multicenter cross-sectional study. Craniomaxillofac Trauma. Reconstr..

[109] De Sousa A. (2008). Psychological issues in acquired facial trauma. Indian. J. Plast. Surg..

